# Editorial: What is esports performance?

**DOI:** 10.3389/fspor.2024.1538686

**Published:** 2024-12-16

**Authors:** David Ekdahl, Ivo van Hilvoorde, Zuzanna Aleksandra Rucińska, Susanne Ravn

**Affiliations:** ^1^ Department of Public Health, Aarhus University, Aarhus, Denmark; ^2^ Department of Behavioural and Movement Sciences, VU Amsterdam, Amsterdam, Netherlands; ^3^ Centre for Philosophical Psychology, University of Antwerp, Antwerp, Belgium; ^4^ Department of Sports Science and Biomechanics, University of Southern Denmark, Odense, Denmark

**Keywords:** esports, sports, digital sport, olympics, embodided cognition, physicality, training, esports career

**Editorial on the Research Topic**
What is esports performance?

The rapid growth of esports underscores its increasing need to be taken seriously by commerce, nations and sports organisations. Born from and rooted in the digital age, esports furthermore manifests ideas and ways of being engaged and performing that generate a wealth of novel questions for research and academic discussion. Esports has thus unsurprisingly emerged as a rich subject of academic inquiry, with researchers exploring whether esports is suitably physical to be considered sport (1–5), whether esports could become an Olympic discipline (6), but also esports specific issues relating to health (7), gender (8–10), spectatorship (11–13), economics (14–16), matters of governance, management and institutionalization (17–21), as well as different ethical issues rapidly emerging within and around esports (22–26). Researchers have moreover taken an active interest in esports as novel kinds of bodily practices (27–30), with esports performances serving as potentially illuminating cases for broader discussions within cognitive science on the relationship between cognition, embodiment and other virtual technologies (31–38).

With this research topic, held together by original and methodologically diverse contributions from fifteen authors, the aim is to add further nuance to the phenomenon of esports performances.

Starting from an often-debated feature of esports performance, namely its hybrid, virtual-physical nature (5, 39, 40) Stapley et al. contextualize the hybrid case of the *Arena Games Triathlon* (ATG). As a competitive Triathlon platform, partially borne out of the Covid-19 pandemic, ATG virtualizes the cycling and running elements of the competitive sport by integrating these physical performances with digital platforms. The authors explore and discuss both the ATG-specific practical challenges in incorporating virtual platforms for competitive cycling and running, as well as the opportunities afforded in doing so. This includes ATG's high media engagement (compared to the World Triathlon) and the unique training- and evaluation-specific opportunities that virtualized and competitive platforms offer. Notably, as a novel injection into the ongoing debate about esports' relationship to the Olympics (6, 41), the authors further contemplate the possibilities for ATG as a hybrid phenomenon fitting into the future Olympics-based esports initiatives.

Staying on the topic of esports' hybridity, Østergaard et al. turn to the impact of physical activity on esports performance. In this regard, the relationship between physical activity and esports performance has long interested researchers (42–45). Specifically, with an eye towards younger players (for whom traditional endurance- and strength-training practices might resonate comparatively less) the authors focus on *playful* forms of bodily activity as a potentially relevant feature of esports training. In this regard, through interviews with and observations of relevant players and coaches, the authors demonstrate the significance of playful activities for the participants' esports performance. Notably, the authors further find that the comparatively more valuable, playful activities deployed tend to relate to and resonate with the particular esports platforms played by the participants. The study's results thus add further nuance to existing conceptual and empirical research, pointing to a close relationship between esports performance, play and embodiment (27, 28, 30, 46–48).

Looking to illuminate esports coverage, Rong & Li’s study explores the role of esports insiders with regard to off-season, player transfers. The authors examine the different competitive structures of different esports practices, before turning to the complex dynamics of the esports transfer market. Focusing on the esports platform CS:GO, the authors analyse data collected from the Perfect World Esports APP and HLTV websites. Identifying an increase in the popularity of transfer information since 2019, the authors' results show that insiders play a progressively important role in the overall transfer ecology of the competitive platform. Touching upon dynamics not always apparent, the authors thus provide a more detailed picture of this platform's transfer ecology, as well as some of its distinct social dynamics.

With two distinct contributions, Rogers et al. turn their attention specifically to first-person shooters in esports contexts. Their first contribution moves from recognising the lack of game-based metrics for quantitative assessments of esports shooting performances to a positive assessment of one possible software for gauging precisely this kind of esports performance, namely the aim trainer *KovaaK's*. In their second study, operationalizing Kovaak's aim trainer as a performance gauge, the authors investigate the impact of caffeine intake on esports performance specifically in the context of first-person shooters. Their results point to a correlation between caffeine intake and increased player precision and reaction time. What is noteworthy here is that the authors find no significant difference in KovaaK's-specific performative effects between higher doses (3 mg·kg^−1^ BM) and lower doses (1 mg·kg^−1^ BM) of caffeine. Given the prevalence of caffeine consumption amongst esports players, both amateur and professional (49–51), these results might contribute to a more informed caffeine ingestion culture in esports performance, specifically amongst younger players. Furthermore, the study (and future similar studies) are urgently needed to inform the debate on the use of performance enhancing drugs in esports, and the limits that should be set for certain substances, related to the specific kind of performances and training in esports.

We hope those interested in esports performance find value in the original contributions of this Research Topic, and that the ideas, issues and suggestions raised by the authors are brought to bear on future esports discourse, initiatives and research.
